# Supplementary material for: Chromothripsis during telomere crisis is independent of NHEJ, and consistent with a replicative origin
Source: Genome Res. 2019 May;29(5):737–49. doi: 10.1101/gr.240705.118 (PMC6499312; doi:10.1101/gr.240705.118)
Supplement: Supplemental Material [file supp_gr.240705.118_Supplemental_file_1.zip › contigs/annotated_contigs/DB78/contig.2.DB78_length_500_mean_cov_4.404.docx]

**DB78_length_500_mean_cov_4.404**

```
TTGTTTCCATTGTTTTTGTTTACAAAAGCTAATCCTTGTTACTAAGATAAAACAGAAAGATAAGTAAACTTGGAAACTGACCACTGTGC
 >chr13:85264281-85264683 + E=1e-230
CACTTTGCAAATTGAGAAGTTGAGATGGAAGGTTTTTTCGGGCTAGGGTTAAAAAAAGTCAGTGTTGGTAAAGAGAATACATCAAGGCC

TATTAATAATCTACTTCTTGTGTTTCTTGACATTACAATTCTACATTGTATCTTATCCAGAGCCAAAGTGCTTCTATATAGCCACTACC

TAATGAATGAATTTACAAGAAAAAAAAACAAAGCAAAGCAAAAGCAACCAGAAACTGATGATCCACCTGAAACTCAAGGATTTCCTATA

ATCATGGACCTCTTTTGATTTGGAAACCTGGATAAAGATGAC|AATT|AGCTGGGTGTGGTGGCACATGCCTATACTCCCAGGTACTCG
                                           >chr13:85270097-85270199 + E=9e-50
GGAGGCTGAGGCAGGGGAATTGCTTGAACTCCGCAGGTGGAGGTTGCAGTGAGCCAA
```
